# Tobacco Use, Exposure to Secondhand Smoke, and Training on Cessation Counseling Among Nursing Students: Cross-Country Data from the Global Health Professions Student Survey (GHPSS), 2005–2009

**DOI:** 10.3390/ijerph6102534

**Published:** 2009-09-28

**Authors:** Charles W. Warren, Dhirendra N. Sinha, Juliette Lee, Veronica Lea, Nathan R. Jones

**Affiliations:** 1 Office on Smoking and Health, US Centers for Disease Control and Prevention, Atlanta, GA, 30341 USA; E-Mails: jpa7@cdc.gov (J.L.); vcl7@cdc.gov (V.L.); 2 Tobacco Free Initiative, South East Asia Region, World Health Organization, Delhi, India; E-Mail: sinhad@searo.who.int; 3 Paul P. Carbone Comprehensive Cancer Center, University of Wisconsin, Madison, WI 53705 USA; E-Mail: nrjones@uwcarbone.wisc.edu

**Keywords:** tobacco use, health professionals, nursing students, counseling training

## Abstract

The Nursing Global Health Professions Student Survey (GHPSS) has been conducted in schools in 39 countries and the Gaza Strip/West Bank (identified as “sites” for the remainder of this paper). In half the sites, over 20% of the students currently smoked cigarettes, with males having higher rates than females in 22 sites. Over 60% of students reported having been exposed to secondhand smoke in public places in 23 of 39 sites. The majority of students recognized that they are role models in society, believed they should receive training on counseling patients to quit using tobacco, but few reported receiving any formal training. Tobacco control efforts must discourage tobacco use among health professionals, promote smoke free workplaces, and implement programs that train health professionals in effective cessation-counseling techniques.

## 1. Introduction

Tobacco use is one of the major preventable causes of premature death and disease in the world [1]. A disproportionate share of the global tobacco burden falls on developing countries, where 84% of the estimated 1.3 billion current smokers reside [1]. The World Health Organization (WHO) attributes approximately five million deaths a year to tobacco. The number is expected to exceed eight million deaths by 2030, with approximately 70% of these deaths occurring in developing countries [2].

Nurses have been found to play an important role in cessation and prevention of tobacco use among their patients [3–6]. Counseling by nurses has been shown to increase smoking cessation [3]. Despite the involvement of nurses, as the largest group of healthcare professionals in tobacco control, only a few studies have collected information on tobacco use, exposure to secondhand smoke, and training to provide cessation counseling among nursing students. These studies used different sampling methods, questionnaires, and data collection procedures, and very few are from low or middle-income countries [7–10]. The WHO, U.S. Centers for Disease Control and Prevention, and the Canadian Public Health Association have attempted to overcome these limitations by developing and implementing the Global Health Professions Student Survey (GHPSS) [11]. GHPSS includes surveys of dental, medical, nursing and pharmacy students. The data reported in this study come from Nursing GHPSS conducted among 3^rd^ year nursing students in 39 countries and the Gaza Strip/West Bank (identified as “sites” for the remainder of this paper) and measures their tobacco use, exposure to secondhand smoke, school policy and enforcement regarding smoking bans, and attitudes toward and training in patient smoking cessation counseling.

## 2. Methods

### 2.1. Design

The Nursing GHPSS is part of the Global Tobacco Surveillance System, which collects data through four surveys: the Global Youth Tobacco Survey, the Global School Personnel Survey, the Global Adult Tobacco Survey, and GHPSS. GHPSS is a school-based survey of 3rd year students pursuing advanced degrees in dentistry, medicine, pharmacy, and nursing. GHPSS uses a core questionnaire on demographics, prevalence of cigarette smoking and use of other tobacco products, exposure to secondhand smoke (SHS), desire to quit smoking, and training received to provide patient counseling on cessation techniques. GHPSS has a standardized methodology for selecting participating schools and uniform data processing procedures [11].

The Nursing GHPSS included a census of students and schools in all locations; except in Armenia, Bosnia and Herzegovina (BiH), Serbia, Bolivia, Brazil, Peru, India, Thailand, and South Korea, where a sample of schools was selected with probability proportional to size from all nursing schools in the country and a census of students in the selected schools were surveyed. The Nursing GHPSS was conducted in schools during regular lectures and class sessions. Anonymous, self-administered data collection procedures were used. Where appropriate, the English questionnaire was translated to native languages then back-translated to English to check for accuracy. SUDAAN, a software package for statistical analysis of complex survey data, was used to calculate weighted prevalence estimates and standard errors (SE) of the estimates (95% confidence intervals (CI) were calculated from the SEs) [12]. For all sites that conducted a census a finite population correction factor was applied to take into account non-response and used in the variance of the estimates. T-tests were used to determine differences between subpopulations [13,14]. In this paper, differences in proportions are considered statistically significant if the t-test p value was less than 0.05.

For sites conducting the Nursing GHPSS, the school response rate was 100% in 33 of the 40 sites, the class room response rate was 100% in all sites, the student response rate ranged from less than 50% (Iran and Armenia) to 100% (Costa Rica), and the overall response rate ranged from 38.2% to 100% (Table 1). The number of students who participated in each survey varied due to the number of schools and students in each sample design.

### 2.2. Measurement

This report includes information on current cigarette smoking defined as those who smoked cigarettes on one or more days in the past 30 days, current use of tobacco products other than cigarettes, exposure to SHS at home and in public places, and the extent to which schools have official policies banning smoking in school buildings and clinics, and if the policies are enforced. In addition, attitude questions were asked regarding: health professionals as role models for their patients, whether health professionals think they should get training in patient cessation techniques, and if they have ever received formal training on such cessation counseling techniques. The final country questionnaires were translated into local languages as needed and back-translated to check for accuracy.

Results in this report are presented by WHO region with select countries highlighted. The six WHO regions are the African Region (AFR), the Eastern Mediterranean Region (EMR), the European Region (EUR), the Americas Region (AMR), the South East Asian Region (SEAR), and the Western Pacific Region (WPR).

## 3. Results

### 3.1. Student Characteristics

The percentage of nursing students who were females ranged from 53.0% (Iraq) to over 80% in 26 sites (Table 2). Over 50% of the students were less than age 25 in every site except Uganda, Jordan, Argentina, Brazil, Cuba, and Trinidad & Tobago.

### 3.2. Tobacco Use

For current cigarette use, less than 10% of nursing students currently smoked cigarettes in all four AFR sites; males were significantly more likely to smoke than females in Kenya and Uganda (Table 3). Current cigarette smoking ranged from 43.9% (Jordan) to less than 5% (Iran and Sudan) in EMR; males were significantly more likely than females to smoke in all 8 EMR sites. In EUR, current cigarette smoking ranged from over 30% in seven of the 10 sites to less than 10% in Armenia and Kyrgyzstan; males were significantly more likely to smoke than females in Albania, Armenia, and Slovakia. In AMR, current cigarette smoking was over 20% in seven of the 11 sites and less than 10% in Jamaica, Panama, and Trinidad & Tobago. There was no gender difference in current smoking in four of the 11 sites; males had higher smoking than females in Costa Rica, Cuba, Peru, and Trinidad & Tobago; and females had higher smoking than males in Chile and Uruguay. Current cigarette smoking was less than 5% in all four SEAR sites; however, males had higher smoking rates than females in all sites. In WPR, current cigarette smoking ranged from 19.9% in Mongolia to less than 4% in Cambodia and South Korea, males were significantly more likely to smoke than females in Cambodia and Mongolia.

Among nursing students, less than 10% currently used other tobacco products in all four AFR sites; however males were significantly more likely than females to use other tobacco products in Algeria and Kenya (Table 3). In the eight EMR sites, other tobacco use was over 20% in Gaza Strip/West Bank and Lebanon but less than 10% in Iran, Iraq, and Sudan; males were significantly more likely than females to use other tobacco products in all 8 EMR sites. In EUR, other tobacco use was 10% or less in all 10 sites; there was no gender difference in six of the 10 sites, males had higher use than females in Serbia and Slovakia, and females had higher use than males in Kyrgyzstan. In AMR, use of other tobacco products was less than 10% in all 11 sites; females had higher use than males in Argentina, Panama, and Uruguay, males had higher use than females in Trinidad & Tobago, and there was no gender difference in five sites. Use of other tobacco products was less than 10% in all four SEAR sites, and males had higher use rates than females in Bangladesh, India, and Sri Lanka. In WPR, use of other tobacco products ranged from 16.4% in Mongolia to less than 4% in Cambodia and South Korea, males had higher use than females in Mongolia.

### 3.3. Exposure to Secondhand Smoke (SHS)

Over 70% of the students reported that they had been exposed to SHS in their home in the past seven days in seven of the 40 sites; compared to less than 40% in 17 sites (Table 4). Less than 50% of the students in all four AFR sites reported exposure to SHS at home in the past seven days; whereas exposure at home was greater than 50% in six of eight sites in EMR (less than 40% in Iran and Sudan), greater than 50% in six of 10 sites in EUR (less than 40% in Czech Republic), greater than 50% in four of 11 sites in AMR (less than 40% in six sites), less than 40% in all four SEAR sites, and in WPR half of the students reported exposure to SHS at home in Cambodia and Mongolia but only 24% in South Korea.

Over 60% of the students reported that they had been exposed to SHS in public places in the past seven days in 23 of the 39 sites; compared to less than 50% in four sites (Table 4). Exposure to SHS in public places was greater than 60% in one of four sites in AFR; greater than 60% in five of seven sites in EMR (reaching 83.0% in Lebanon); greater than 60% in nine of 10 sites in EUR (over 80% in four sites); greater than 60% in five of 11 sites in AMR (over 80% in five sites); greater than 60% in one of four SEAR sites; and greater than 60% in two of the three WPR sites (Table 4).

The proportion of students reporting their schools have an official policy banning smoking in school buildings and clinics was over 60% in 15 of the 39 sites; and less than 40% in 15 sites (Table 4). Having the policy was less likely in EMR (all seven sites reported less than 40%) than the other regions. Over 70% of the students reported enforcement of the policy in 19 of the 39 sites. Enforcement was less than 30% in Iraq, Tunisia, Albania, and Brazil.

### 3.4. Health Professional Roles and Training

Over 70% of the students thought health professionals have a role in giving advice about smoking cessation to patients in 37 of 38 sites, with 19 over 90% (Table 5). The lowest was in Slovakia (57.4%). Over 90% of the students thought health professionals should get specific training on cessation techniques in 30 of the 39 sites; with the lowest in Iraq (65.0%) and Czech Republic (66.5%). Less than 40% of the students reported having ever received some kind of formal training in their professional school on cessation approaches to use with their patients in 30 of the 39 sites. Over half of the students had received formal training in only four sites (Iraq, Sudan, Kyrgyzstan, and Republic of Moldova). In seven of the 39 sites, less than 20% of the students had received the training.

## 4. Discussion

Findings from the Nursing GHPSS show that over 20% of nursing students currently smoke cigarettes in 19 of 40 sites; over 40% in four sites (Jordan, Albania, Chile, and Uruguay). Among the six WHO regions, current cigarette smoking was highest in EMR, EUR and AMR based on sites that have completed the Nursing GHPSS. Males were more likely than females to smoke cigarettes in 22 of 38 sites; females had higher rates than males in Chile and Uruguay. Use of other forms of tobacco was over 10% in six of 40 sites and over 40% in Lebanon. Among the WHO regions, use of other tobacco products was highest in EMR, probably reflecting the high use of waterpipe (Shisha) in the region. Males were more likely than females to use other tobacco products in 18 of 38 sites; females had a higher rate than males in Argentina, Ghana, Kyrgyzstan, Panama, Uganda and Uruguay. Tobacco use endangers the health of nursing students and negatively influences the future nursing workforce to deliver effective anti-tobacco counseling when they start seeing patients [9]. The tobacco control community should target tobacco users among nursing students to overcome this situation. Educational institutions training nurses should help their students quit using tobacco by providing encouragement and information to students who are considering quitting and providing assistance to students who are motivated to quit.

Over 60% of nursing students reported they were exposed to SHS in public places in 23 of the 39 sites. However, in 15 of the 39 sites over 60% of the students reported their schools have an official policy banning smoking in school buildings and clinics. Enforcement of the school policies is very high. Educational institutions training nurses should be encouraged to provide smoke free work and study areas by banning smoking in their buildings and clinics. A smoke free work environment has been shown to improve air quality, reduce health problems associated with exposure to tobacco smoke, support and encourage cessation attempts among smokers trying to quit, and receive high levels of public support from people who spend time in the area. [15] Furthermore, the creation of smoke free areas by health education institutions sends a clear message to educators, students, patients, and clinicians about negative impact of tobacco. [16]

Nursing students should be trained to provide effective, accurate, and accessible advice to patients on all aspects of health. Nursing GHPSS data show that over 70% of nursing students recognize that they are role models in society (in 37 of 38 sites), over 90% think they should receive training on counseling and treating patients to quit using tobacco (29 of 39 sites), but less than 40% have received formal training in 30 of 39 sites.

The Nursing GHPSS surveyed 3rd year students, so it is possible that students receive training on patient cessation techniques during the latter years of their programs. To address this possibility, the GHPSS research coordinators raised this question to the school administrators and found that, in the majority of the countries, there is no formal training at any time. Of the countries with some training, the type of training included: problem-based learning, included in generic counseling curricula; or included in curricula as part of community medicine or public health courses. This study did not make an effort to evaluate the adequacy of cessation training in the countries reporting this type of instruction. However, professional training for nursing students should include courses detailing the harmful health effects of tobacco use and exposure to secondhand smoke, and training in counseling on tobacco cessation techniques [4–7,17,18]. Curricula should include a course or supplements to existing courses specifically relevant to tobacco issues. If administrators are resistant to making changes in the core curricula, schools should be encouraged to incorporate tobacco-related modules within existing courses.

The majority of evaluation research conducted on tobacco-related curricula has been conducted in high income countries. Relatively little information about the process of teaching nursing students in low and middle-income countries about smoking prevention and cessation is accessible to the international tobacco control community. Peer-reviewed studies in international settings about educational materials and techniques to improve the capacity of nurses to treat and counsel patients on cessation are necessary to focus limited resources on effective and efficient strategies to reduce the prevalence of tobacco use. Efforts should be made to assess and share the content of tobacco control components within the formal training curricula and continuing education courses for nursing students. Further research should be carried out to assess the impact of existing tobacco control-related materials and training provided in nursing schools in a variety of cultural and economic environments. The products from such research could form a compendium of “best practices” of patient counseling for training nurses relevant to countries with a broad spectrum of health resources and infrastructures.

## 5. Conclusions

Educational institutions, public health organizations, and education officials should discourage tobacco use among nurses and work together to design and implement programs that train nurses in effective cessation-counseling techniques. The Nursing GHPSS has shown significant unmet need for cessation assistance among nursing students as well as gaps in professional training to provide similar effective assistance to their future patients. The Nursing GHPSS is helpful in evaluating the behavior and attitudes regarding tobacco among nursing students, but additional research is necessary to improve the evidence base for effective tobacco-related curricula, especially materials that are appropriate for a range of cultural and economic settings. If the goal of the tobacco control community is to reduce substantially the use of tobacco products, then resources should be invested in improving the quality of education of nurses with respect to tobacco control.

## What this paper adds

- An alarming proportion of nursing students currently smoked cigarettes and used other tobacco products. Although the majority of nursing students believed that health professionals should receive training to assist patients with tobacco cessation, only a small proportion of students have received such training.
- The Nursing GHPSS has shown significant unmet need for cessation assistance among nursing students as well as gaps in professional training to provide similar effective assistance to their future patients.

## Figures and Tables

**Table 1.** Response rates by region and country, Nursing Global Health Professions Student Survey, 2005–2009.

**Country (Site)**	**Year**	**School Response Rate (%)**	**Class Response Rate (%)**	**Student Response Rate (%)**	**Overall Response Rate (%)**	**Number of 3rd Year Students**
**AFRICAN REGION (AFR)**						
Algeria	2007	100.0	100.0	68.4	68.4	167
Ghana	2006	100.0	100.0	81.0	81.0	133
Kenya	2008	100.0	100.0	95.4	95.4	148
Uganda	2005	100.0	100.0	94.1	94.1	395
**EASTERN MEDITERRANEAN REGION (EMR)**						
Gaza Strip/West Bank	2007	100.0	100.0	95.5	95.5	208
Iran	2007	88.9	100.0	43.0	38.2	1162
Iraq	2005	100.0	100.0	93.2	93.2	54
Jordan	2007	100.0	100.0	99.6	99.6	775
Lebanon	2006	100.0	100.0	68.3	68.3	343
Sudan	2007	100.0	100.0	83.1	83.1	284
Syrian Arab Republic	2006	100.0	100.0	94.7	94.7	989
Tunisia	2007	100.0	100.0	68.2	68.2	374
**EUROPEAN REGION (EUR)**						
Albania	2005	100.0	100.0	68.2	68.2	338
Armenia	2006	100.0	100.0	42.0	42.0	506
Bosnia and Herzegovina	2005	100.0	100.0	86.0	86.0	855
Czech Republic	2006	81.8	100.0	86.0	70.4	348
Greece	2009	100.0	100.0	74.5	74.5	187
Kyrgyzstan	2008	100.0	100.0	77.4	77.4	159
Lithuania	2006	100.0	100.0	76.3	76.3	303
Republic of Moldova	2008	100.0	100.0	89.3	89.3	275
Serbia	2006	91.7	100.0	88.2	80.9	2069
Slovakia	2006	100.0	100.0	90.2	90.2	405
**REGION OF THE AMERICAS (AMR)**						
Argentina	2007	100.0	100.0	93.2	93.2	269
Bolivia	2006	100.0	100.0	99.3	99.3	602
Brazil (Rio de Janeiro)	2006	90.0	100.0	76.4	68.8	954
Chile	2008	94.3	100.0	80.1	75.6	1490
Costa Rica	2006	100.0	100.0	100.0	100.0	156
Cuba (Havana)	2008	100.0	100.0	78.7	78.7	255
Jamaica	2008	100.0	100.0	88.5	88.5	211
Panama	2008	100.0	100.0	87.9	87.9	292
Peru	2006	95.5	100.0	95.6	91.3	1238
Trinidad and Tobago	2008	100.0	100.0	86.9	86.9	352
Uruguay	2008	100.0	100.0	99.1	99.1	194
**SOUTH-EAST ASIA REGION (SEAR)**						
Bangladesh	2008	100.0	100.0	90.3	90.3	948
India	2007	100.0	100.0	93.0	93.0	947
Sri Lanka	2006	100.0	100.0	89.7	89.7	443
Thailand	2006	100.0	100.0	88.9	88.9	1594
**WESTERN PACIFIC REGION (WPR)**						
Cambodia	2005	100.0	100.0	91.9	91.9	215
Mongolia	2007	100.0	100.0	95.2	95.2	298
South Korea	2006	95.0	100.0	79.3	75.3	806

**Table 2.** Population characteristics by region and country, Nursing Global Health Professions Student Survey, 2005–2009.

**Country (Site)**	**Year**	**Census or Sample**	**% Female**	**Age 24 and Under**	**Age 25 – 29**	**Age 30+**
**AFRICAN REGION (AFR)**						
Algeria	2007	Census	83.7%	86.0%	11.1%	2.9%
Ghana	2006	Census	78.3%	52.6%	11.7%	35.7%
Kenya	2008	Census	65.0%	93.9%	4.7%	1.5%
Uganda	2005	Census	84.0%	42.3%	25.2%	32.4%
**EASTERN MEDITERRANEAN REGION (EMR)**						
Gaza Strip/West Bank	2007	Census	59.2%	96.9%	2.0%	1.0%
Iran	2007	Census	82.3%	97.9%	1.9%	0.3%
Iraq	2005	Census	53.0%	90.7%	9.3%	0.0%
Jordan	2007	Census	60.4%	NA	NA	NA
Lebanon	2006	Census	74.2%	95.4%	4.0%	0.7%
Sudan	2007	Census	80.7%	59.2%	2.4%	38.4%
Syrian Arab Republic	2006	Census	70.4%	95.5%	4.5%	0.1%
Tunisia	2007	Census	73.1%	54.9%	45.1%	0.0%
**EUROPEAN REGION (EUR)**						
Albania	2005	Census	78.8%	91.5%	5.6%	2.8%
Armenia	2006	Sample	91.2%	99.1%	0.6%	0.3%
Bosnia and Herzegovina	2005	Sample	72.8%	99.9%	0.0%	0.1%
Czech Republic	2006	Census	97.6%	95.3%	4.1%	0.5%
Greece	2009	Census	86.6%	93.5%	1.1%	5.4%
Kyrgyzstan	2008	Census	65.8%	91.2%	8.2%	0.6%
Lithuania	2006	Census	90.7%	74.1%	10.8%	15.1%
Republic of Moldova	2008	Census	89.8%	98.7%	0.7%	0.7%
Serbia	2006	Sample	84.0%	100.0%	0.0%	0.0%
Slovakia	2006	Census	94.8%	84.6%	15.3%	0.0%
**REGION OF THE AMERICAS (AMR)**						
Argentina	2007	Census	80.4%	32.3%	17.3%	50.3%
Bolivia	2006	Sample	89.6%	77.5%	17.2%	5.2%
Brazil (Rio de Janeiro)	2006	Sample	85.2%	46.3%	21.8%	32.0%
Chile	2008	Census	86.8%	76.2%	14.4%	9.5%
Costa Rica	2006	Census	79.7%	81.2%	9.1%	9.7%
Cuba (Havana)	2008	Census	92.8%	10.5%	10.9%	78.5%
Jamaica	2008	Census	96.1%	54.5%	24.1%	21.4%
Panama	2008	Census	88.0%	83.8%	10.2%	6.0%
Peru	2006	Sample	84.5%	87.6%	9.3%	3.2%
Trinidad and Tobago	2008	Census	91.5%	39.7%	27.7%	32.6%
Uruguay	2008	Census	87.0%	63.8%	26.4%	9.8%
**SOUTH-EAST ASIA REGION (SEAR)**						
Bangladesh	2008	Census	92.0%	94.9%	4.7%	0.4%
India	2007	Sample	87.4%	99.0%	1.0%	0.0%
Sri Lanka	2006	Census	89.6%	74.9%	24.9%	0.2%
Thailand	2006	Sample	93.8%	96.8%	1.9%	1.3%
**WESTERN PACIFIC REGION (WPR)**						
Cambodia	2005	Census	64.8%	93.9%	6.1%	0.0%
Mongolia	2007	Census	78.9%	83.8%	8.4%	7.7%
South Korea	2006	Sample	95.1%	94.2%	4.5%	1.2%

**Table 3.** Prevalence of current tobacco use, by sex, region and country, Nursing Global Health Professions Student Survey 2005–2009.

**Country (Site)**	**Year**	**Current cigarette smokers**				**Currently use other tobacco products**			
		**Total % (95% CI)**	**Male % (95% CI)**	**Female % (95% CI)**	**P–Value**	**Total % (95% CI)**	**Male % (95% CI)**	**Female % (95% CI)**	**P–Value**
**AFRICAN REGION (AFR)**									
Algeria	2007	2.4 (1.1–4.9)	8.5 (3.0–22.2)	1.3 (0.4–3.7)	0.1078	2.3 (1.1–4.7)	9.8 (4.1–21.4)	0.8 (0.2–3.4)	0.0317
Ghana	2006	0.8 (0.3–2.0)	0.0	0.0	NA	1.5 (0.7–2.9)	0.0	1.9 (1.0–3.8)	0.0040
Kenya	2008	7.5 (6.0–9.3)	13.5 (10.3–17.6)	4.3 (3.0–6.3)	0.0000	5.4 (4.2–7.0)	9.7 (7.0–13.3)	3.1 (2.0–4.8)	0.0002
Uganda	2005	0.5 (0.3–0.9)	3.3 (1.9–5.6)	0.0	0.0004	0.8 (0.5–1.2)	0.0	0.9 (0.6–1.4)	0.0000
**EASTERN MEDITERRANEAN REGION (EMR)**									
Gaza Strip/West Bank	2007	25.0 (23.6–26.5)	33.9 (31.5–36.5)	19.9 (18.2–21.6)	0.0000	24.8 (23.4–26.2)	30.2 (27.9–32.7)	20.1 (18.4–21.8)	0.0000
Iran	2007	4.4 (3.4–5.6)	17.4 (13.1–22.6)	1.6 (1.0–2.5)	0.0000	8.8 (7.4–10.3)	22.6 (17.9–28.3)	5.7 (4.6–7.2)	0.0000
Iraq	2005	18.7 (15.8–22.1)	31.8 (26.6–37.5)	7.4 (4.9–11.0)	0.0000	5.5 (4.0–7.7)	7.9 (5.3–11.7)	3.6 (2.0–6.5)	0.0288
Jordan	2007	43.9 (43.1–44.8)	62.2 (61.1–63.2)	16.0 (15.0–17.0)	0.0000	16.4 (15.8–17.1)	22.6 (21.7–23.5)	7.4 (6.7–8.1)	0.0000
Lebanon	2006	26.9 (24.2–29.7)	43.0 (37.1–49.1)	21.5 (18.7–24.7)	0.0000	44.9 (41.8–48.0)	54.3 (48.3–60.1)	41.6 (38.1–45.2)	0.0003
Sudan	2007	4.8 (3.7–6.2)	21.6 (16.5–27.7)	0.9 (0.4–1.7)	0.0000	3.7 (2.8–5.0)	12.4 (8.6–17.5)	1.3 (0.7–2.3)	0.0000
Syrian Arab Republic	2006	19.3 (18.7–20.0)	49.8 (48.4–51.2)	7.0 (6.6–7.5)	0.0000	19.0 (18.4–19.6)	32.5 (31.2–33.8)	13.5 (12.8–14.1)	0.0000
Tunisia	2007	26.2 (23.4–29.1)	57.9 (51.4–64.1)	14.7 (12.2–17.6)	0.0000	19.1 (16.7–21.8)	37.7 (32.0–43.8)	12.1 (9.7–14.9)	0.0000
**EUROPEAN REGION (EUR)**									
Albania	2005	41.5 (37.9–45.1)	57.5 (49.8–64.8)	36.4 (32.5–40.5)	0.0000	1.5 (0.9–2.4)	2.1 (0.9–4.7)	1.3 (0.7–2.3)	0.4161
Armenia	2006	5.7 (2.9–10.8)	48.6 (30.9–66.7)	2.4 (0.8–6.8	0.0051	1.1 (0.5–2.6)	2.7 (1.1–6.7)	1.0 (0.3–3.7)	0.2540
Bosnia and Herzegovina	2005	33.0 (28.8–37.6)	27.3 (21.1–34.5)	34.8 (29.8–40.2)	0.0707	5.5 (4.1–7.4)	7.0 (3.8–12.4)	5.0 (3.4–7.3)	0.4043
Czech Republic	2006	32.7 (29.7–35.8)	*	33.2 (30.1–36.5)	NA	4.3 (3.2–5.9)	*	4.3 (3.1–5.9)	NA
Greece	2009	33.5 (30.0–37.3)	40.0 (30.2–50.7)	32.5 (28.8–36.6)	0.1882	2.7 (1.7–4.3)	4.1 (1.4–11.2)	2.5 (1.5–4.2)	0.4739
Kyrgyzstan	2008	9.5 (7.5–12.0)	9.3 (6.1–13.8)	9.7 (7.2–12.9)	0.8510	9.6 (7.6–12.1)	5.6 (3.2–9.4)	11.9 (9.1–15.3)	0.0043
Lithuania	2006	36.2 (33.1–39.3)	32.5 (24.1–42.2)	36.6 (33.3–39.9)	0.4082	7.3 (5.8–9.1)	10.4 (5.8–17.8)	6.9 (5.4–8.9)	0.2713
Republic of Moldova	2008	20.2 (16.2–24.8)	28.0 (15.1–45.8)	19.4 (15.3–24.2)	0.2998	7.6 (5.2–11.0)	10.6 (3.9–25.7)	6.9 (4.5–10.5)	0.4896
Serbia	2006	33.8 (27.2–41.1)	32.4 (22.5–44.3)	34.1 (28.1–40.7)	0.5205	10.0 (6.9–14.4)	13.4 (8.9–19.6)	9.4 (6.3–13.7)	0.0416
Slovakia	2006	32.2 (30.6–33.8)	41.8 (34.8–49.2)	31.7 (30.1–33.3)	0.0077	4.3 (3.7–5.0)	15.0 (10.3–21.2)	3.7 (3.1–4.4)	0.0001
**REGION OF THE AMERICAS (AMR)**									
Argentina	2007	36.4 (33.3–39.6)	38.4 (31.5–45.8)	36.0 (32.5–39.6)	0.5510	7.7 (6.1–9.7)	1.9 (0.7–5.4)	9.3 (7.3–11.7)	0.0000
Bolivia	2006	21.3 (8.8–43.3)	36.8 (13.3–68.8)	19.5 (9.2–36.7)	0.0549	9.1 (1.1–48.3)	14.9 (3.1–48.6)	8.4 (0.9–47.4)	0.1107
Brazil (Rio de Janeiro)	2006	12.5 (8.4–18.2)	10.8 (4.1–25.3)	12.9 (8.8–18.6)	0.5795	4.0 (2.0–8.1)	4.6 (1.0–19.4)	3.9 (1.6–9.0)	0.8250
Chile	2008	46.6 (45.2–47.9)	40.2 (36.7–43.8)	47.6 (46.1–49.0)	0.0002	3.0 (2.6–3.5)	2.4 (1.5–3.8)	3.1 (2.6–3.6)	0.2860
Costa Rica	2006	24.0	25.8	23.3	NA	5.8	9.7	4.1	NA
Cuba (Havana)	2008	39.8 (36.9–42.7)	62.5 (51.4–72.4)	38.2 (35.2–41.2)	0.0000	7.6 (6.2–9.3)	6.1 (2.4–14.5)	7.8 (6.3–9.6)	0.5755
Jamaica	2008	5.1 (4.0–6.5)	*	5.3 (4.2–6.8)	NA	2.1 (1.5–3.1)	*	1.8 (1.2–2.6)	NA
Panama	2008	3.4 (2.7–4.3)	3.2 (1.5–6.6)	3.5 (2.7–4.4)	0.8229	2.2 (1.6–2.9)	0.0	2.5 (1.8–3.3)	0.0000
Peru	2006	25.0 (21.7–28.7)	42.0 (34.6–49.9)	22.0 (18.5–25.8)	0.0001	4.7 (3.1–7.2)	7.4 (3.1–16.7)	3.9 (2.4–6.1)	0.2355
Trinidad and Tobago	2008	5.7 (4.8–6.7)	16.1 (11.7–21.7)	4.8 (3.9–5.8)	0.0000	1.1 (0.8–1.7)	3.0 (1.4–6.3)	1.0 (0.6–1.5)	0.0000
Uruguay	2008	41.9 (39.2–44.8)	23.9 (17.8–31.1)	44.7 (41.6–47.7)	0.0000	6.9 (5.6–8.5)	0.0	8.0 (6.5–9.9)	0.0000
**SOUTH-EAST ASIA REGION (SEAR)**									
Bangladesh	2008	4.0 (3.6–4.5)	49.5 (45.6–53.5)	0.3 (0.2–0.5)	0.0000	8.1 (7.5–8.7)	26.4 (23.1–29.9)	6.5 (6.0–7.1)	0.0000
India	2007	3.4 (1.9–5.9)	19.9 (10.3–35.1)	1.1 (0.3–3.3)	0.0071	4.5 (3.0–6.7)	14.5 (7.7–25.7)	2.9 (1.5–5.6)	0.0175
Sri Lanka	2006	1.0 (0.7–1.4)	7.6 (5.3–10.7)	0.3 (0.1–0.5)	0.0000	2.8 (2.3–3.4)	17.9 (14.3–22.1)	1.1 (0.8–1.5)	0.0000
Thailand	2006	1.1 (0.6–2.3)	9.8 (4.4–20.3)	0.5 (0.2–1.5)	0.0211	1.0 (0.6–1.6)	4.4 (1.3–14.2)	0.7 (0.3–1.6)	0.1635
**WESTERN PACIFIC REGION (WPR)**									
Cambodia	2005	4.3 (3.6–5.2)	12.3 (10.3–14.6)	0.0	0.0000	0.0	0.0	0.0	NA
Mongolia	2007	19.9 (18.8–20.9)	53.9 (51.0–56.8)	11.0 (10.1–11.9	0.0000	16.5 (15.6–17.5)	36.6 (33.9–39.3)	11.1 (10.2–12.1)	0.0000
South Korea	2006	4.2 (2.7–6.7)	13.1 (4.1–34.8)	3.6 (2.3–5.6)	0.1839	3.1 (1.1–8.4)	18.0 (6.0–43.1)	2.2 (0.9–4.9)	0.0608

**Table 4.** Exposure to secondhand smoke (at home and in public places) and school policy and enforcement regarding bans on smoking, region and country, Nursing Global Health Professions Student Survey, 2005–2009.

**Country (Site)**	**Year**	**In the past 7 days, had someone smoke in their presence and their home**	**In the past 7 days, had someone smoke in their presence other than in their home**	**Have an official policy banning smoking in school buildings and clinics**	**Have an official policy banning smoking in school buildings and clinics and the policy is enforced**
		**Total % (95% CI)**	**Total % (95% CI)**	**Total % (95% CI)**	**Total % (95% CI)**
**AFRICAN REGION (AFR)**					
Algeria	2007	29.3 (24.3–35.0)	45.8 (40.0–51.7)	62.8 (56.9–68.4)	41.2 (33.8–49.0)
Ghana	2006	23.9 (20.5–27.6)	37.5 (33.6–41.7)	49.8 (45.6–54.0)	57.6 (51.6–63.4)
Kenya	2008	47.7 (44.6–50.9)	63.9 (60.8–66.9)	61.1 (57.7–64.4)	76.4 (72.3–80.0)
Uganda	2005	30.4 (28.6–32.3)	52.5 (50.5–54.5)	23.8 (21.9–25.8)	79.3 (75.1–82.9)
**EASTERN MEDITERRANEAN REGION (EMR)**					
Gaza Strip/West Bank	2007	59.5 (57.9–61.1)	68.4 (66.8–69.8)	37.3 (35.8–38.9)	86.8 (84.7–88.5)
Iran	2007	37.0 (34.5–39.5)	55.8 (53.2–58.4)	30.1 (27.8–32.6)	69.7 (65.0–74.0)
Iraq	2005	57.4 (53.4–61.3)	64.9 (61.0–68.6)	28.3 (24.8–32.1)	28.1 (21.6–35.6)
Jordan	2007	81.0 (80.4–81.7)	NA	NA	NA
Lebanon	2006	74.1 (71.3–76.8)	83.0 (80.5–85.2)	36.5 (33.6–39.6)	63.2 (58.0–68.0)
Sudan	2007	35.2 (32.4–38.1)	55.4 (52.4–58.4)	21.4 (19.0–24.0)	78.7 (72.7–83.7)
Syrian Arab Republic	2006	74.2 (73.5–74.8)	76.9 (76.2–77.5)	30.6 (29.9–31.3)	64.7 (63.4–66.0)
Tunisia	2007	53.8 (50.6–57.1)	64.5 (61.3–67.6)	37.8 (34.6–41.1)	25.8 (21.0–31.3)
**EUROPEAN REGION (EUR)**					
Albania	2005	79.2 (76.3–81.8)	93.8 (92.0–95.3)	24.1 (20.7–27.7)	29.5 (22.7–37.5)
Armenia	2006	67.2 (60.6–73.2)	60.2 (53.4–66.7)	23.3 (18.2–29.3)	91.6 (85.1–95.4)
Bosnia and Herzegovina	2005	77.1 (74.4–79.5)	85.7 (82.7–88.4)	44.2 (39.1–49.3)	63.8 (54.8–71.8)
Czech Republic	2006	38.9 (35.8–42.2)	87.6 (85.3–89.6)	89.6 (87.4–91.4)	67.6 (64.2–70.8)
Greece	2009	54.7 (50.8–58.6)	66.0 (62.2–69.6)	27.5 (24.2–31.2)	32.0 (25.3–39.7)
Kyrgyzstan	2008	40.3 (36.6–44.0)	41.4 (37.7–45.2)	32.3 (28.8–36.0)	39.4 (31.6–47.8)
Lithuania	2006	46.1 (42.9–49.2)	71.3 (68.5–74.0)	53.5 (50.3–56.6)	41.3 (37.1–45.6)
Republic of Moldova	2008	47.9 (42.6–53.1)	74.5 (69.6–78.9)	55.5 (50.1–60.7)	97.7 (94.0–99.2)
Serbia	2006	78.8 (73.9–83.0)	91.3 (89.4–92.8)	44.4 (31.5–58.2)	81.4 (68.3–89.9)
Slovakia	2006	53.3 (51.7–55.0)	76.0 (74.5–77.4)	88.3 (87.2–89.4)	67.2 (65.4–68.9)
**REGION OF THE AMERICAS (AMR)**					
Argentina	2007	52.8 (49.6–56.1)	82.5 (79.9–84.9)	48.7 (45.5–52.0)	68.5 (64.0–72.6)
Bolivia	2006	38.5 (27.1–51.5)	58.4 (46.4–69.5)	31.5 (21.5–43.6)	70.0 (52.9–82.9)
Brazil (Rio de Janeiro)	2006	38.1 (33.2–43.2)	80.1 (73.3–85.6)	3.8 (1.4–10.2)	11.7 (5.5–23.0)
Chile	2008	50.7 (49.4–52.0)	84.4 (83.4–85.4)	66.0 (64.5–67.4)	54.3 (52.3–56.3)
Costa Rica	2006	44.9	85.2	74.8	72.8
Cuba (Havana)	2008	79.8 (77.4–82.0)	88.5 (86.5–90.2)	58.0 (55.0–60.8)	42.8 (38.9–46.7)
Jamaica	2008	28.1 (25.6–30.7)	56.7 (53.9–59.5)	77.9 (75.4–80.2)	78.5 (75.7–81.0)
Panama	2008	26.2 (24.3–28.1)	57.2 (55.0–59.3)	47.1 (44.9–49.3)	58.5 (55.1–61.7)
Peru	2006	33.8 (30.7–37.1)	59.4 (54.8–63.9)	37.5 (29.2–46.6)	72.4 (60.6–81.7)
Trinidad and Tobago	2008	33.2 (31.3–35.1)	59.4 (57.4–61.4)	73.6 (71.7–75.4)	74.6 (72.4–76.7)
Uruguay	2008	52.0 (49.1–54.8)	46.1 (43.3–48.9)	84.7 (82.6–86.7)	82.5 (80.0–84.7)
**SOUTH-EAST ASIA REGION (SEAR)**					
Bangladesh	2008	27.6 (26.7–28.5)	55.0 (54.0–56.1)	81.0 (80.1–81.8)	71.1 (70.0–72.2)
India	2007	32.2 (25.6–39.5)	50.8 (43.3–58.2)	68.1 (58.5–76.3)	86.4 (82.7–89.4
Sri Lanka	2006	17.1 (15.8–18.4)	63.4 (61.4–65.2)	77.2 (75.9–78.5)	93.7 (92.7–94.6)
Thailand	2006	28.6 (24.6–32.9)	59.6 (53.8–65.2)	66.4 (61.0–71.4)	95.5 (93.5–97.0)
**WESTERN PACIFIC REGION (WPR)**					
Cambodia	2005	50.9 (49.0–52.8)	51.5 (49.5–53.4)	64.2 (62.4–66.1)	72.8 (70.4–75.1)
Mongolia	2007	49.1 (47.8–50.4)	66.8 (65.6–68.0)	59.0 (57.8–60.3)	68.4 (66.8–70.1)
South Korea	2006	23.9 (20.9–27.1)	70.8 (60.7–79.2)	61.7 (52.1–70.5)	71.0 (58.4–81.0)

**Table 5.** Attitudes toward and training in patient smoking cessation counseling, region and country, Nursing Global Health Professions Student Survey, 2005–2009.

**Country (Site)**	**Year**	**Think health professionals have a role in giving advice or information about smoking cessation to patients**	**Think health professionals should get specific training on cessation techniques**	**Have ever received any formal training in smoking cessation approaches to use with patients in their nursing school training**
		**Total % (95% CI)**	**Total % (95% CI)**	**Total % (95% CI)**
**AFRICAN REGION (AFR)**				
Algeria	2007	74.5 (69.0–79.4)	95.7 (92.6–97.6)	46.7 (40.9–52.6)
Ghana	2006	98.5 (97.1–99.3)	98.5 (97.1–99.3)	18.8 (15.8–22.3)
Kenya	2008	98.6 (97.7–99.2)	100.0	27.7 (24.9–30.6)
Uganda	2005	98.4 (97.8–98.9)	97.1 (96.3–97.7)	35.1 (33.2–37.0)
**EASTERN MEDITERRANEAN REGION (EMR)**				
Gaza Strip/West Bank	2007	89.5 (88.5–90.5)	93.5 (92.6–94.2)	38.4 (36.8–40.0)
Iran	2007	88.8 (87.0–90.4)	96.3 (95.3–97.1)	13.3 (11.7–15.0)
Iraq	2005	72.3 (68.5–75.7)	65.0 (61.1–68.7)	56.4 (52.3–60.4)
Jordan	2007	NA	NA	NA
Lebanon	2006	85.4 (83.1–87.4)	96.4 (94.9–97.4)	43.7 (40.6–46.8)
Sudan	2007	98.9 (98.0–99.4)	98.3 (97.3–99.0)	53.6 (50.6–56.6)
Syrian Arab Republic	2006	98.2 (97.9–98.4)	97.3 (97.0–97.5)	30.6 (29.9–31.3)
Tunisia	2007	84.0 (81.5–86.2)	93.8 (91.9–95.2)	45.6 (42.3–48.9)
**EUROPEAN REGION (EUR)**				
Albania	2005	89.4 (87.2–91.4)	96.7 (95.3–97.7)	22.6 (20.0–25.4)
Armenia	2006	83.1 (80.0–85.8)	89.5 (81.3–94.3)	42.1 (18.8–69.5)
Bosnia and Herzegovina	2005	NA	90.3 (87.8–92.3)	28.6 (23.7–34.0)
Czech Republic	2006	71.6 (68.5–74.5)	66.5 (63.3–69.6)	7.4 (5.9–9.3)
Greece	2009	96.7 (95.0–97.9)	95.3 (93.4–96.7)	14.0 (11.5–17.0)
Kyrgyzstan	2008	83.0 (80.0–85.7)	82.4 (79.3–85.1)	57.6 (53.8–61.3)
Lithuania	2006	86.7 (84.0–89.1)	96.9 (95.4–97.9)	33.7 (30.6–36.9)
Republic of Moldova	2008	87.5 (83.5–90.6)	82.2 (77.9–85.9)	67.3 (62.2–72.1)
Serbia	2006	88.2 (86.9–89.4)	78.9 (75.1–82.3)	38.7 (33.0–44.8)
Slovakia	2006	57.4 (55.7–59.2)	71.2 (69.7–72.8)	11.1 (10.1–12.2)
**REGION OF THE AMERICAS (AMR)**				
Argentina	2007	76.1 (73.2–78.7)	94.4 (92.7–95.7)	15.3 (13.1–17.8)
Bolivia	2006	88.8 (75.1–95.5)	98.5 (91.9–99.7)	37.5 (14.7–67.6)
Brazil (Rio de Janeiro)	2006	73.2 (57.8–84.4)	95.2 (93.1–96.7)	27.6 (17.6–40.5)
Chile	2008	96.9 (96.4–97.3)	94.0 (93.4–94.6)	25.7 (24.6–26.9)
Costa Rica	2006	91.7	96.1	12.8
Cuba (Havana)	2008	100.0	96.6 (95.4–97.5)	24.0 (21.6–26.6)
Jamaica	2008	99.1 (98.5–99.5)	97.0 (95.9–97.8)	23.7 (21.4–26.1)
Panama	2008	100.0	98.6 (98.0–99.1)	35.7 (33.6–37.8)
Peru	2006	92.7 (90.8–94.2)	99.1 (97.9–99.6)	25.5 (19.6–32.5)
Trinidad and Tobago	2008	97.4 (96.6–98.0)	93.1 (92.0–94.1)	20.2 (18.6–21.8)
Uruguay	2008	91.0 (89.2–92.5)	94.8 (93.4–95.9)	24.0 (21.7–26.5)
**SOUTH-EAST ASIA REGION (SEAR)**				
Bangladesh	2008	98.9 (98.6–99.1)	95.9 (95.5–96.3)	43.2 (42.2–44.3)
India	2007	96.7 (95.7–97.5)	90.1 (87.9–91.9)	35.1 (27.2–43.9)
Sri Lanka	2006	84.4 (83.3–85.5)	77.7 (76.3–78.9)	21.0 (19.6–22.3)
Thailand	2006	91.0 (89.2–92.5)	94.8 (93.4–95.9)	24.0 (21.7–26.5)
**WESTERN PACIFIC REGION (WPR)**				
Cambodia	2005	99.5 (99.2–99.7)	98.6 (98.1–99.0)	29.9 (28.2–31.7)
Mongolia	2007	79.5 (78.4–80.5)	89.8 (89.0–90.6)	24.5 (23.4–25.6)
South Korea	2006	96.0 (90.2–98.4)	87.6 (85.8–89.2)	37.9 (29.6–46.9)

## GHPSS Country Research Coordinators

**WHO Regional Offices**: African Region, Jean-Pierre Baptiste, Nivo Ramanandraibe; Eastern Mediterranean Region, Fatimah El-Awa, Heba Fouad; European Region, Agis Tsouros, Kristina Mauer-Stender, Rula Nabil Khoury; Region of the Americas, Adriana Blanco, Roberta Caixeta; South-East Asia Region, Khalilur Rahman, Dhirendra N. Sinha; Western Pacific Region, Susan Mercado, Ali Akbar.

**African Region:** Djamel Zoughailech (Algeria); Edith Wellington, (Ghana); Joyce Nato, Ezra Ouma Ogwell (Kenya); Frederick Musoke (Uganda).

**Eastern Mediterranean Region:** Samah Eriqat, Salah Shaker Isa Soubani, Moein Al Kariry (Gaza Strip and West Bank); Ahmed Ali Bahaj, Ali Asghar Farshad, Hassan Azaripour Masooleh (Iran); Sarhang Jalal (Iraq); Heba Ayoub, Imam Al Jaghbeer (Jordan); Georges Saade, Nagib Ghosn (Lebanon); Ibrahim Abdelmageed Mohamed Ginawi, Ilham Abdalla Bashir (Sudan); Bassam Abu Al Zahab (Syria); Mohamed Nabil Ben Sahem, Alya Mahjoub Zarrouk, Mohamed Mokdad, Mongi Hamrouni (Tunisia).

**European Region:** Roland Shuperka (Albania); Alexander Bazarjyan (Armenia); Aida Ramic-Catak, Zivana Gavric (Bosnia & Herzegovina); Hana Sovinova (Czech Republic); Elpidoforos Soteriades (Greece); Aisha Tokobaeva (Kyrgyzstan); Antanas Gostautas (Lithuania); Vorfolomei Calmic (Republic of Moldova); Djordje Stojilkovic, Andjelka Dzeletovic (Republic of Serbia); Tibor Baska (Slovakia).

**Region of the Americas:** Hugo A. Miguez (Argentina); Franklin Alcaraz del Castillo (Bolivia); Luisa Goldfarb, Valeska Caralho Figueiredo, Adelemara Mattoso Allonzi, Leticia Casado Costa, Liz Maria de Almeida (Brazil); Claudia Gonzalez Wedmaier (Chile); Julio Bejarano (Costa Rica); Lucia Lances Cotilla (Cuba); Ellen Grizzle (Jamaica); Reina Roa (Panama); Alfonso Zavaleta (Peru); Leo Alleyne, Nicole Cooper (Trinidad & Tobago); Raquel Magri, Gabriela Olivera (Uruguay).

**South-East Asia Region:** Zulfiqar Ali (Bangladesh); Prakash C Gupta, Mangesh Pednekar, Dhirendra N. Sinha (India); P. W. Gunasekera (Sri Lanka); Nithat Sirichotiratana (Thailand).

**Western Pacific Region:** Sin Sovann, Sung Vin Tak (Cambodia); Dondog Jargalsaikhan, L. Erdenebayar, Palam Enkhtuya, Tsogzolmaa Bayandorj (Mongolia); Sun Ha Jee (South Korea). CDC Support: Michelle Carlberg, Ann Goding, Brandon M. O’Hara
